# A “Swiss paradox” in the United States? Level of spatial aggregation changes the association between income inequality and morbidity for older Americans

**DOI:** 10.1186/s12942-019-0192-x

**Published:** 2019-11-27

**Authors:** Steven A. Cohen, Mary L. Greaney, Ann C. Klassen

**Affiliations:** 1 Department of Health Studies, University of Rhode Island, 25 West Independence Way, Suite P, Kingston, RI 02881 USA; 2 Dornsife School of Public Health, Drexel University, 3215 Market Street, Room 455, Philadelphia, PA 19104 USA

## Abstract

Although a preponderance of research indicates that increased income inequality negatively impacts population health, several international studies found that a greater income inequality was associated with better population health when measured on a fine geographic level of aggregation. This finding is known as a “Swiss paradox”. To date, no studies have examined variability in the associations between income inequality and health outcomes by spatial aggregation level in the US. Therefore, this study examined associations between income inequality (Gini index, GI) and population health by geographic level using a large, nationally representative dataset of older adults. We geographically linked respondents’ county data from the 2012 Behavioral Risk Factor Surveillance System to 2012 American Community Survey data. Using generalized linear models, we estimated the association between GI decile on the state and county levels and five population health outcomes (diabetes, obesity, smoking, sedentary lifestyle and self-rated health), accounting for confounders and complex sampling. Although state-level GI was not significantly associated with obesity rates (b = − 0.245, 95% CI − 0.497, 0.008), there was a significant, negative association between county-level GI and obesity rates (b = − 0.416, 95% CI − 0.629, − 0.202). State-level GI also associated with an increased diabetes rate (b = 0.304, 95% CI 0.063, 0.546), but the association was not significant for county-level GI and diabetes rate (b = − 0.101, 95% CI − 0.305, 0.104). Associations between both county-level GI and state-level GI and current smoking status were also not significant. These findings show the associations between income inequality and health vary by spatial aggregation level and challenge the preponderance of evidence suggesting that income inequality is consistently associated with worse health. Further research is needed to understand the nuances behind these observed associations to design informed policies and programs designed to reduce socioeconomic health inequities among older adults.

## Background

The relationship between income and health is well established, with a higher income being indicative of greater health. This association extends to a variety of health measures, including, but not limited to, general health status [1, 2], health care access [3], obesity [4, 5], cancer [6, 7], diabetes [8], as well as health outcomes linked to environmental conditions [9, 10], and summary measures of population health [11–13].

Numerous studies have determined that higher income inequality, the difference within a group between those with the highest and lowest incomes, negatively impacts population health above and beyond the contribution of low income itself [14–20]. Income inequality is associated with many differences in social determinants of health at the individual- and population level that may adversely influence health status and behaviors [21]. Numerous hypotheses exist about how increased income inequality leads to or contributes to poorer health outcomes. Kawachi and Kennedy posited that the observed associations between income inequality and health may be, in part, due to underinvestment in social goods, such as public education and health care, as well as eroding social cohesion and social capital [14, 22]. Areas of high-income inequality may promote lower levels of societal trust, and that may exacerbate the deterioration of social cohesion, which, in turn, promotes worsening health outcomes [20, 23]. The effect of income inequality and health outcomes among older adults is especially notable, with higher income inequality being associated with an increased prevalence of depression among older adults in the United States (US) [24] and Mexico [25]. A cross-national study that compared mortality rate changes across age groups over time suggests that income inequality may be a driver for mortality to a greater extent among older adults than younger adults [26].

In sharp contrast to existing studies, however, Clough-Gorr et al. [27] found that increased income inequality in Switzerland was associated with better health outcomes, including mortality from non-external causes, and they named this finding the “Swiss paradox”. Results from two recent studies in the US [28] and Australia [29] support the idea of a Swiss paradox, while a South African study identified no significant associations between income inequality and mortality and morbidity [30]. Three studies used similar units of spatial aggregation and measured income inequality on a finer geographic level of aggregation than prior studies. The US study [28] used county-level income inequality, a commonly used unit of spatial aggregation, while the Australian study [29] used Local Government Areas (LGAs) as the spatial unit of analysis. LGAs are small subdivisions within each Australian state and territory that generally provide residents services, including infrastructure and other municipal services. LGAs are comparable to cities and towns or county subdivisions in the US [31]. Similarly, the South African study [30] used districts as the spatial unit of analysis. Districts serve as similar function in the provision of services to its residents. There are 52 total districts in South Africa, with population sizes ranging from just over 74,000 to 5 million (Johannesburg) [32]. Regardless of the country-specific spatial unit used, the explanation for these paradoxical patterns remains unclear.

Few studies have used a life course perspective to examine the cumulative nature of income inequality’s effect, especially on chronic health burden. The US population is aging, with an estimated one in five US adults being 65 years of age or older by 2030 [33]. Health disparities driven by socioeconomic inequalities, such as income and education, are widening [20, 34, 35], particularly among older adults [36–38]. Of the few existing studies, a negative association between national-level income inequality and healthy life expectancy among residents of 33 countries was observed, but the study did not focus on older adults specifically [37].

To date, to our knowledge, no US studies have examined the variability in the associations between income inequality and health by level of geographic aggregation. The few studies that have examined the relationships between income inequality and health, in general, have not specifically examined those associations among older adults. Therefore, the objective of this exploratory study was to compare and contrast the associations between several measures of population health and income inequality on two geographic levels of aggregation, the state and the county, among older adults in the US using a large, national database.

## Methods

### Data sources and sample

The study sample included respondents aged 65–99 drawn from the 2012 Behavioral Risk Factor Surveillance System (BRFSS) public-use data set. The BRFSS, administered by the Centers for Disease Control and Prevention (CDC), is the largest system of health-related telephone surveys in the US, with approximately 500,000 interviews conducted annually with US residents in all 50 states aged 18+. Respondents report their demographic characteristics, household income, health-related risk behaviors, height, weight, chronic health conditions, and use of screening and preventive services [39]. The results of analyses from BRFSS are used for planning and prevention efforts.

This study used the 2012 BRFSS sample, the most recent year in which respondent’s place of residence (county) was collected. It should be noted that county size and function vary across states and regions in the US. The 2012 BRFSS included 475,687 respondents, with a 49.1% response rates for landline and a 35.3% rate for cell phones [40]. The analytic sample for the current study was restricted to respondents aged 65+ who were living in the contiguous US (lower 48 states), because county of residence was not available in the 2012 BRFSS for residents of Alaska and Hawaii. The resultant sample size was 152,541 (32.1%). After the final sample was selected, each respondent was linked to area-level data from the 2010 US Census via county Federal Information Processing Standard (FIPS) code.

### Measures

#### Key predictor variables

The two key predictor variables were county and state-level measures of the Gini index, a common measure of income inequality used in public health studies [41–46]. The Gini index is measured on a scale from 0 to 1, with 0 indicating perfect income equality, and 1 indicating perfect income inequality. Therefore, the higher the Gini index, the higher the income inequality is in a given area. The Gini indices were directly obtained from the 2010 US Census [47] and American Community Survey [48] for both the county and state of residence for each BRFSS respondent included in the current study. Gini indices were recoded as Gini index deciles at the county and state levels in the analysis to facilitate interpretation [49–51].

#### Outcome measures

Five representative health outcomes were examined for this analysis: (1) self-reported health status (SRH), (2) obesity status, (3) diabetes status, (4) sedentary lifestyle, and (5) current smoking status.

1. Self-reported health (SRH) was determined from the question: “Would you say that in general your health is… excellent, very good, good, fair, or poor?” Response options were dichotomized for analysis (fair/poor vs. good/very good/excellent).
2. Obesity status was calculated using self-reported height and weight to determine body mass index (BMI) which was then used to calculate weight status. Respondents with a BMI of 30 kg/m^2^ or above were classified as having obesity.
3. Diabetes status was ascertained through the question “Has a medical professional ever told you that you have diabetes…?” Respondents who answered “yes” were classified as having diabetes.
4. Sedentary lifestyle was determined using a single item: “During the past month, other than your regular job, did you participate in any physical activities or exercises such as running, calisthenics, golf, gardening, or walking for exercise?” Respondents who answered “no” to this question were considered to have a sedentary lifestyle.
5. Current smoking status was assessed by the following question: “Do you now smoke cigarettes every day, some days, or not at all?” Respondents who answered “every day” or “some days” were considered to be current smokers.

#### Confounders and covariates

Several confounders and covariates were examined in this study. Individual level confounders and covariates included: age (65–69, 70–74, 75–79, 80+), sex, race/ethnicity (White, Black, Hispanic, Other), individual income level (in $25,000 increments), and education level (less than college degree, college degree or higher). Two county-level covariates were obtained from the 2010 US Census: population density as a measure of rural–urban status, and per capita income at the county level as a measure of socioeconomic status.

#### Data analysis

The BRFSS data set included the CDC analytic sample weights, which were used in all analyses to account differences in sampling and response probabilities [39, 40]. Descriptive statistics were obtained for all variables, including weighted means and standard deviations or medians and interquartile ranges for all continuous and discrete variables, and weighted frequencies and percentages for all categorical variables. Bivariate associations were obtained through the use of Chi squared tests, t-tests, and Pearson and Spearman correlation coefficients, depending on the variable types. Demographic characteristics and the five examined health outcomes were compared at the county-level Gini index using Chi square statistics.

Respondents were aggregated by their county of residence to obtain weighted county-specific prevalence estimates for each of the five examined health outcomes: poor/fair SRH, obesity, diabetes, lifestyle, and currently smoking. The associations between the key predictor variable, decile of Gini index, and each of the five outcome variables were assessed using generalized linear modeling (GLM) with a linear link function, with separate models constructed for county- and state-level Gini indices. Each health outcome was examined in three models (for a total of 30 models): using the Gini index at both the county and state levels: (1) bivariate models examining the association between each individual outcomes; (2) a set of models with Gini index alone; and (3), a set of bivariate models that adjusted for per capita income and the examined covariates and confounders. The potential for non-normality of the outcome variables and (county-specific prevalence estimates) and model residuals was also explored and a sensitivity analysis also was conducted using natural log-transformed prevalence estimates as the outcome variables. Statistical significance was set at p < 0.05 for all analyses. SPSS version 24 (Armonk, NY) and SAS version 9.4 (Cary, NC) were used for the statistical analyses, and ArcGIS version 10 (Redlands, WA) was used for mapping.

## Results

**Fig. 1:**
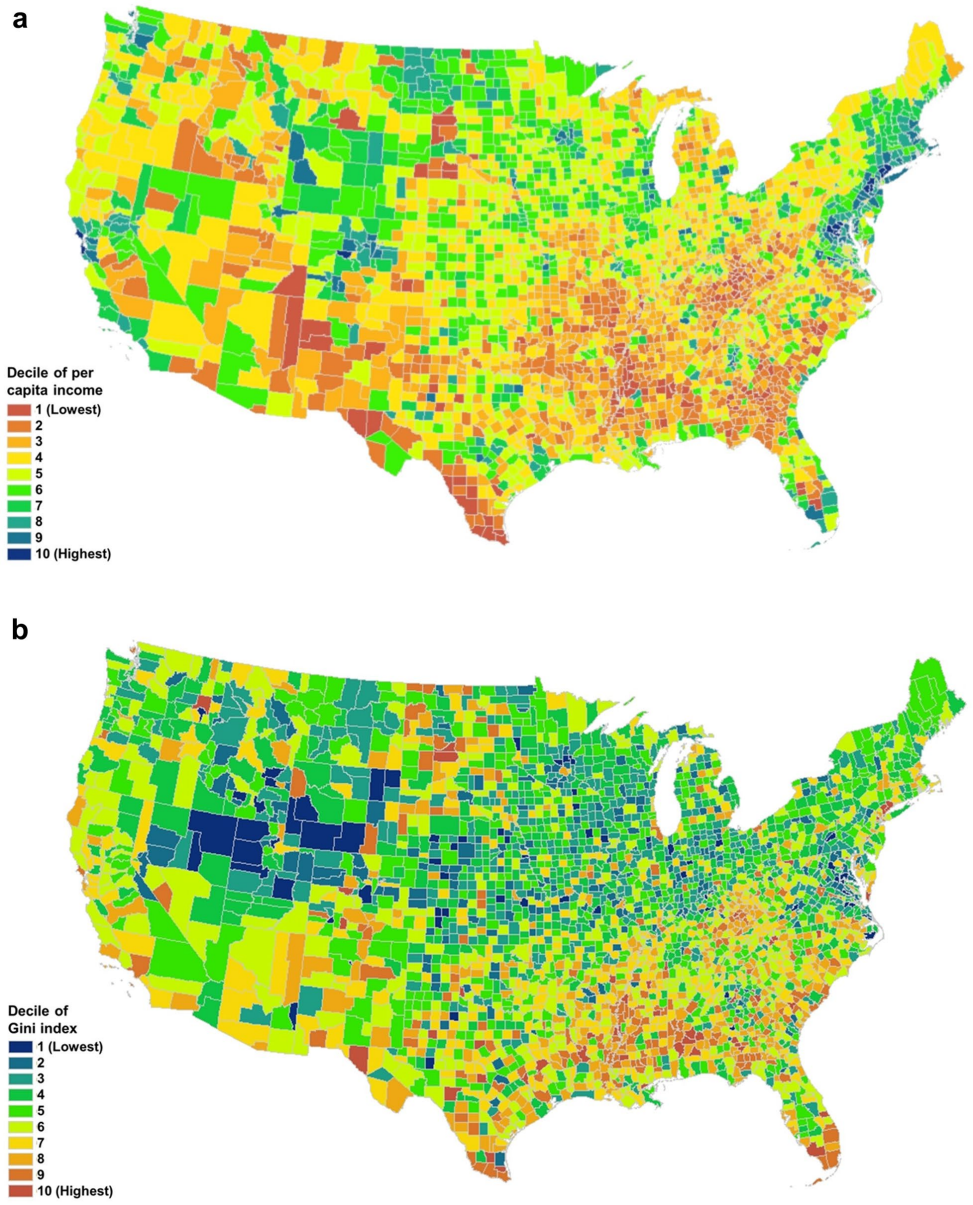

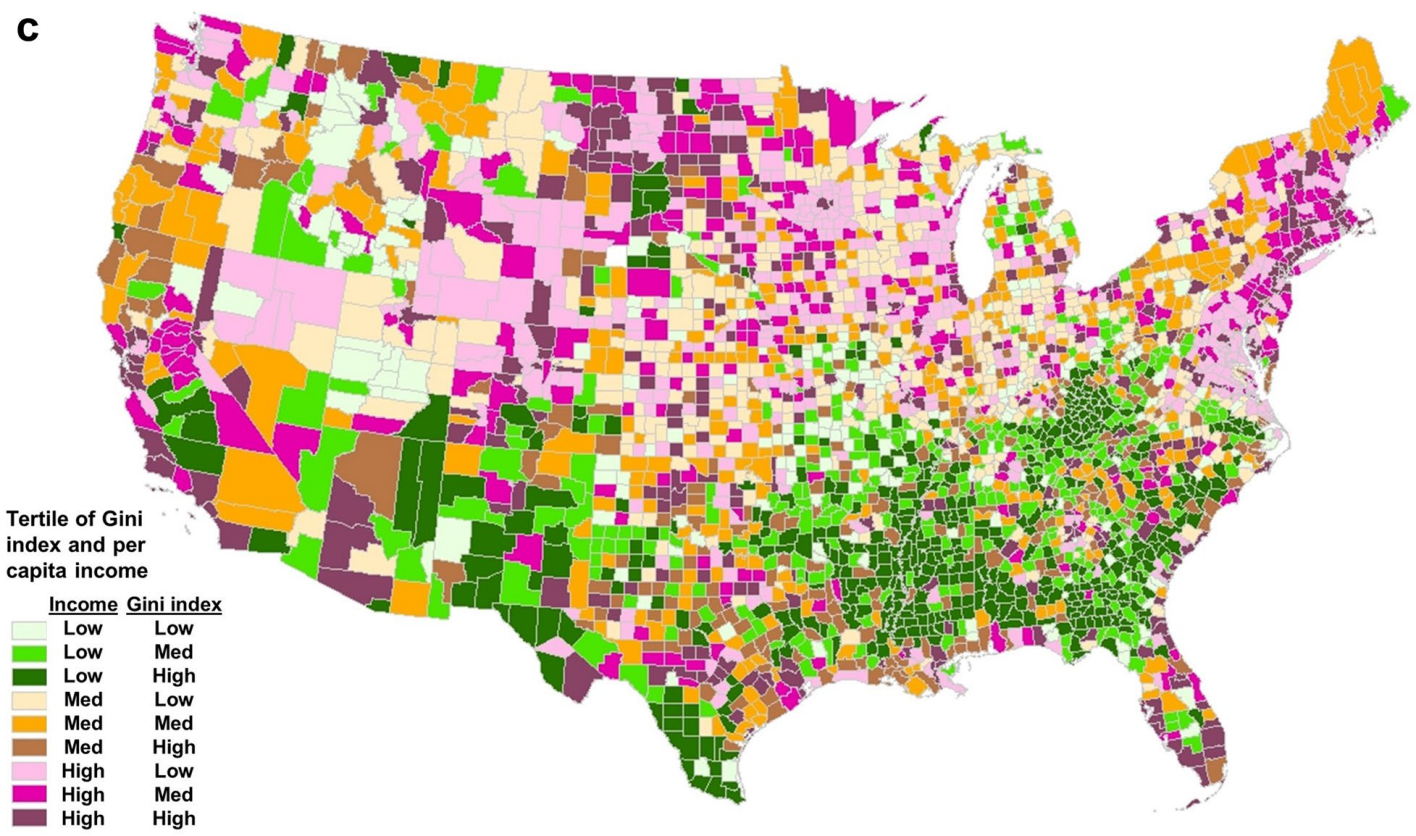
County-level distributions of the Gini index (**a**), per capita income (**b**), and the joint distributions of the Gini index and per capita income (**c**)

**Fig. 2:**
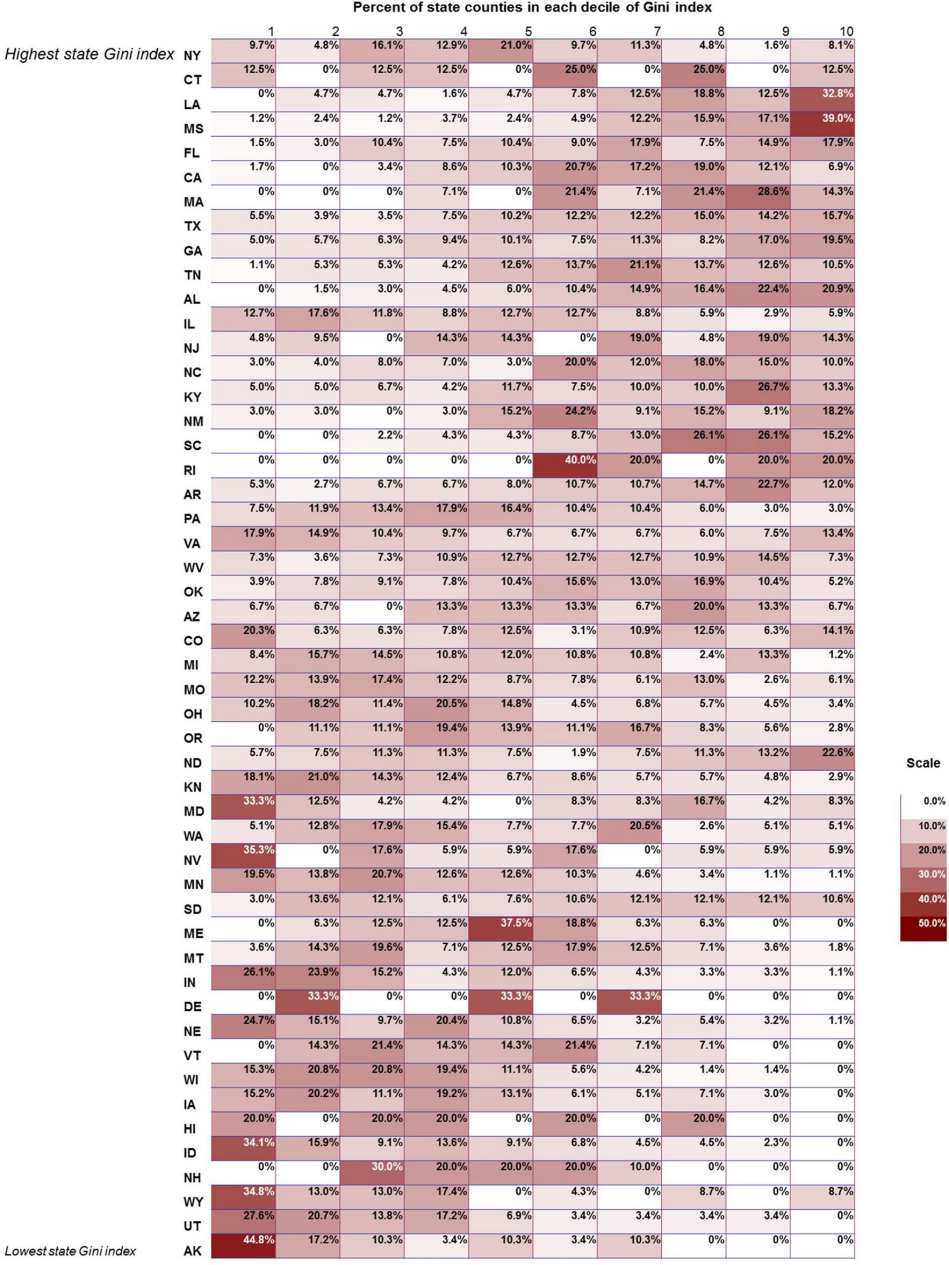
Percent of individual state’s counties by each decile of the Gini index ranked from highest state Gini index (top) to lowest state Gini index (bottom)

Descriptive statistics and frequencies by county-level income inequality above or below the median Gini index (0.389–0.442) are shown in Table 1. Respondents’ average age was 74.5 (± 7.20) years. Respondents living in areas of high income inequality (Gini index 0.442–0.599) were more likely to be female (57.2% vs. 56.0% in low Gini index counties), over 80 years old (24.6% vs. 22.7%), Black (10.9% vs. 4.6%) or Hispanic (8.8% vs. 3.8%), and college graduates (25.1% vs. 19.4%). Figure 1 shows the distribution of the Gini index, per capita income, and the joint distributions of Gini index and per capita income. New York had the highest Gini index while Utah had the lowest (Fig. 2).

**Table 1:** Descriptive statistics for respondents by county-level Gini index (above vs. below median, high vs. low)

Number of counties	1571	1572
	Low Gini (%)	High Gini (%)
Gender		
Male	44.0	42.8
Female	56.0	57.2
Age		
65–69	32.3	32.1
70–74	24.5	23.9
75–79	20.5	19.4
80–99	22.7	24.6
Race		
White	85.9	73.3
Black	4.6	10.9
Hispanic	3.8	8.8
Other	5.7	7.0
Income ($)		
< 25,000	34.9	35.8
25,000–50,000	35.1	31.3
> 50,000	29.9	32.9
College grad		
Yes	19.4	25.1
No	80.6	74.9
Obese		
Yes	27.4	24.5
No	72.6	75.5
Diabetes		
Yes	21.6	21.5
No	78.4	78.5
Poor SRH		
Yes	25.3	26.6
No	74.7	73.4
Sedentary		
Yes	32.3	30.4
No	67.7	69.6
Smoker		
Yes	8.9	8.6
No	91.1	91.4

In terms of the examined health measures, respondents living in counties with a high Gini index were less likely to have obesity (24.5% vs. 27.4%), have a sedentary lifestyle (30.4% vs. 32.3%), or smoke (8.6% vs. 8.9%). They also were more likely to report poor/fair SRH (26.6% vs. 25.3%). The association between Gini index and diabetes status was not significant (p = 0.038).

**Fig. 3:**
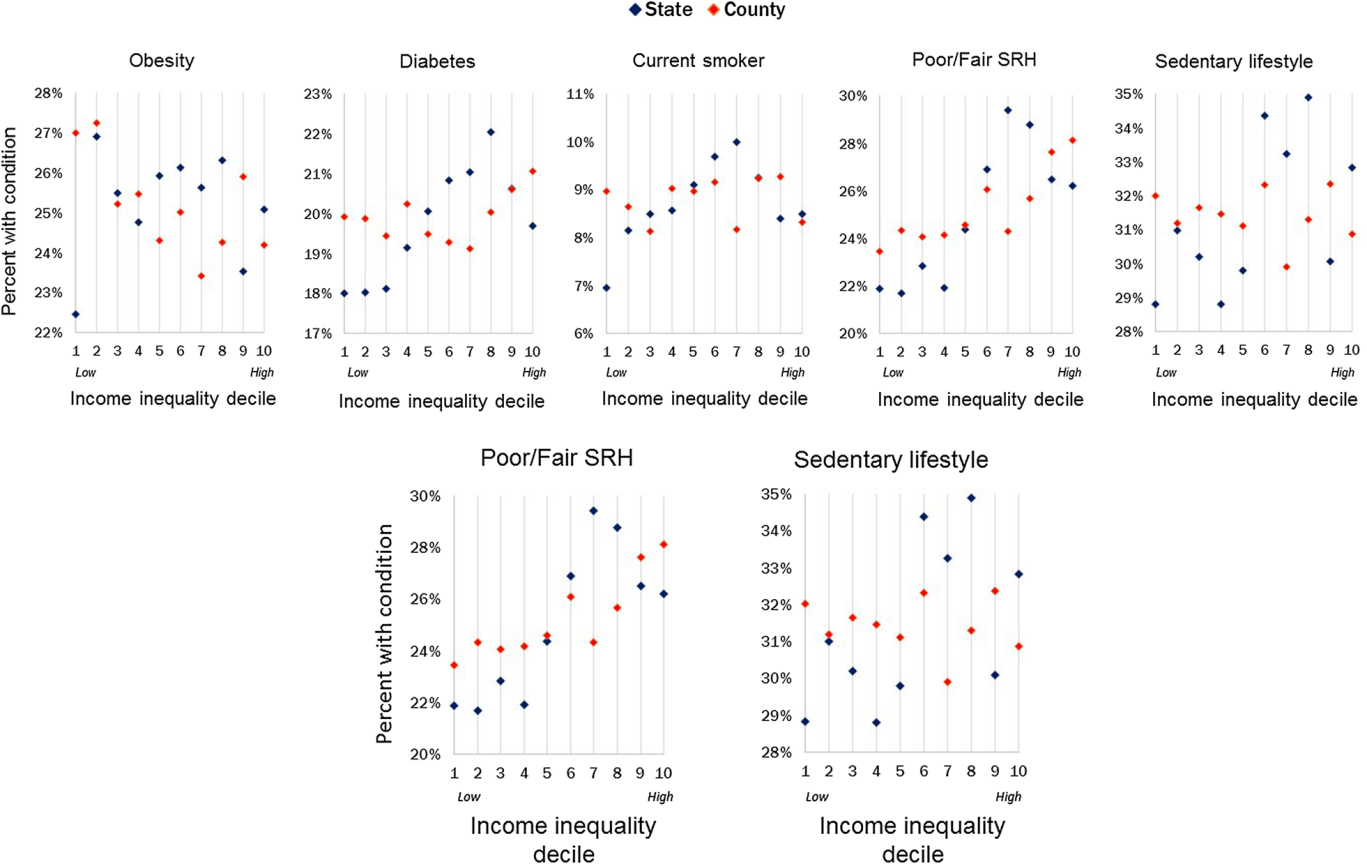
Percent of respondents with each health outcome or behavior by income inequality decile, as measured on the state (blue) and county (red) levels

Figure 3 illustrates the relationships between each of the five health outcome measures and decile of both county and state-level Gini index. Increasing state-level Gini index was correlated with higher levels of poor/fair SRH (r = 0.170, p < 0.001), diabetes (r = 0.100, p < 0.001), sedentary lifestyle (r = 0.101, p < 0.001), and smoking (r = 0.058, p = 0.006), but not with obesity (r = − 0.002, p = 0.923). Increasing county-level income inequality, however, was correlated with lower levels of obesity (r = − 0.068, p = 0.001) and higher levels of poor/fair SRH (r = 0.148, p < 0.001), but not with diabetes, sedentary lifestyle, or current smoking status.

Results of individually modeling each of the five health conditions and behaviors outcomes on decile of state and county-level Gini index are shown in Table 2. Parameter estimates represent the average expected change in the percentage of the health outcome for a one-level increase in the Gini index, county-level or state-level as indicated. Higher levels of income inequality on the county level were associated with a decrease in the prevalence of obesity, even after controlling for potential confounders (b = − 0.416, 95% CI − 0.629, − 0.202). The obesity prevalence and state-level income inequality were not associated in the unadjusted or adjusted models. As level of income inequality at the state level increased, there was a significant increase in the prevalence of diabetes (b = 0.304, 95% CI 0.063, 0.546) and sedentary lifestyle (b = 0.575, 95% CI 0.297, 0.853), which remained, even after adjustment. Neither of these behaviors were significantly associated with county-level income inequality, however.

**Table 2:** Parameter estimates of rate of five health conditions and behaviors based on decile of state and county-level Gini index

Gini level	Obesity	Diabetes	Current smoker	Poor/fair SRH	Sedentary
Unadjusted					
County	*− 0.33 (− 0.54, − 0.13)*	− 0.08 (− 0.12, 0.27)	0.05 (− 0.10, 0.20)	*0.82 (0.59, 1.04)*	0.19 (− 0.04, 0.42)
State	− 0.01 (− 0.25, 0.23)	*0.55 (0.32, 0.78)*	*0.24 (0.07, 0.41)*	*1.11 (0.84, 1.38)*	*0.66 (0.39, 0.94)*
Income-adjusted					
County	*− 0.39 (− 0.59, − 0.19)*	0.03 (− 0.16, 0.23)	0.01 (− 0.14, 0.15)	*0.66 (0.45, 0.88)*	0.05 (− 0.17, 0.28)
State	− 0.09 (− 0.33, 0.15)	*0.50 (0.27, 0.73)*	*0.18 (0.01, 0.36)*	*0.89 (0.64, 1.15)*	*0.48 (0.21, 0.75)*
Fully adjusted^a^					
County	*− 0.42 (− 0.63, − 0.20)*	− 0.10 (− 0.31, 0.10)	0.01 (− 0.14, 0.17)	*0.63 (0.41, 0.86)*	0.23 (− 0.01, 0.47)
State	− 0.25 (− 0.50, 0.01)	*0.30 (0.06, 0.55)*	0.12 (− 0.06, 0.30)	*0.71 (0.44, 0.98)*	*0.58 (0.30, 0.85)*

Italics indicate significant association (p < 0.05)

^a^Fully adjusted models included the following covariates: age (65–69, 70–74, 75–79, 80+), sex, race/ethnicity (White, Black, Hispanic, Other), individual income level (in $25,000 increments), and education level (less than college degree, college degree or higher), county-level population density, and county-level per capita income

The prevalence of poor/fair SRH was significantly and positively associated with income inequality at both the state and county levels, and remained significant after adjusting for confounders (b = 0.633, 95% CI 0.405, 0.861 for county Gini; b = 0.711, 95% CI 0.441, 0.980 for state Gini). Increasing prevalence of smoking was associated with increased income inequality on the state level in both the unadjusted and income-adjusted models, but not in the fully adjusted model. There was no significant association between county-level income inequality and prevalence of smoking for any of the models.

A sensitivity analysis of log-transformed prevalence rates as the outcome variables in the GLM models revealed similar results, except that the association between state-level Gini index and prevalence of smoking became non-significant in the income-adjusted models. Additionally, the association between obesity and state-level Gini index became significant in the fully-adjusted models, where the association was borderline (p = 0.056) in the linear models.

## Discussion

The associations between the Gini index on both the county and state levels and the five selected health outcomes varied, and several notable patterns were identified. First, the prevalence of poor/fair SRH was higher in areas of higher Gini index when measured both on the state and county levels. Increasing state-level Gini index, indicating greater income inequality, was associated with increased prevalence of both diabetes and sedentary lifestyles, whereas, there was no significant association for these outcomes with county-level Gini index. Lastly, there was a significant negative association between the county-level Gini index and the prevalence of obesity, although the association was not significant for the state-level Gini index. There was no significant association for smoking prevalence and the Gini index regardless of whether it was measured on the state or county level after adjustment for confounders.

The findings of this study are potentially important and should be explored in future research and policy in addressing the social determinants of health. In terms of research, these findings emphasize the idea that associations between social determinants and health outcomes may be dependent upon the geographic unit of analysis. Researchers need to carefully consider the level of geographic aggregation when designing studies. For research outside of the US, an important consideration is the availability of reliable data at different geographic levels of aggregation, especially in developing countries [52]. A 2015 study in Sweden demonstrated that ethnically homogenous neighborhoods had higher levels of certain mental health issues compared to more ethnically diverse neighborhoods using the geographic unit of analysis of Small Area Market Statistics (SAMS), each containing an average of 1000 people [53]. The association may not have been observed had a larger level of spatial aggregation been used. Sweden, like many European counties, maintains high quality data on vital statistics and health outcomes that are analyzable on multiple geographic levels. The conclusions of any such study examining place-based social determinants and health outcomes can only be extended to the geographic level of aggregation used.

The policy implications of the study are more nuanced. For example, higher county-level rates of obesity were observed in populations that are more homogeneous with respect to income (i.e. lower income inequality). It is possible that those populations were more homogeneous in terms of other socioeconomic and demographic factors in those local communities. If so, policies could be formulated to address the root causes of obesity that address specific economic, cultural, social, or geographic factors that promote obesity in the most affected counties or areas, such as food deserts, lack of recreational opportunities, infrastructure, etc.

Based on the variability of the associations identified in the current study, increasing income inequality on either the state or county level does not necessarily impart worse health outcomes, with the notable exception of poor/fair SRH. However, importantly, SRH is a reliable predictor of morbidity and mortality in a population [54]. Research suggests that increasing income inequality on the local level (county) may actually be protective against certain health outcomes for older adults, such as obesity. One possible explanation may be that body size is closely influenced by the body size of other members of a person’s social network [55]. Thus, communities that are more homogeneous in terms of income may share collective food cultures and body size norms, including those which promote obesity. Future research should explore these unexpected findings in detail.

These findings are similar to those found by Fan et al. [56], who determined that at the county level, higher income inequality was associated with lower individual risk of obesity for all adults when comparing county-level to census tract-level income inequality. Census tracts are county subdivisions containing approximately 4000 residents and can be thought of as neighborhood equivalents [57]. In this study, the observed magnitude of the association was larger for county-level income inequality than for census tract-level income inequality [56]. Previous research suggests that the opposite is true when examining income inequality on geographic units larger than the county level, such as on the national level [58–60]. For example, a cross-national study showed that higher income inequality was associated with an increased risk of obesity for all adults [58]. Only a few studies have examined state-level income inequality and obesity risk [60, 61]. One study showed a positive relationship between state-level income inequality and cardiovascular outcomes related to obesity: living in a state with higher income inequality was associated with an increased risk for heart attack [62]. State-level income inequality also was positively associated with factors that may cause obesity, such as having a sedentary lifestyle and insufficient physical activity [63]. None of these studies were specific to older adults, the age group with the highest morbidity and mortality rates across the lifespan.

Pickett and Wilkinson [64] evaluated the epidemiological criteria for causality in the association between income inequality and health outcomes and reported that most examined studies that explored income inequality and health status have identified associations between increasing income inequality and worsening population health. Pickett and Wilkinson reported that only a minority of studies have found the opposite association—increased income inequality being associated with better population health outcomes or no association at all [28]. They argue that these findings are less valid than studies examining income inequality on larger geographic scales because assessing income inequality at a finer geographic scale (e.g., county, neighborhood) is not an appropriate measure and determinant of the magnitude of a society’s social stratification. They also suggest the positive relationship between income inequality and population health may be due to factors related to income inequality, namely smaller-scale residential segregation, which may manifest as income inequality.

Although the current exploratory study do not directly assess residential segregation as the cause of income inequality, we suggest an alternative explanation for these findings. Perhaps the unexpected and contradictory findings of higher income inequality being associated with better health outcomes, namely less obesity, on the county level may be due to different mechanisms than those occurring on larger geographic scales. Ignoring inequalities on the community or county level and focusing exclusively on inequities in larger geographic levels may bypass potentially critical points of intervention that may be more effective on the local level than on the larger geographic level [65]. Addressing inequalities on multiple levels may be key to maximizing the effectiveness of any interventions and polities and may capitalize on existing sociocultural dynamics and infrastructure that may be driving potentially positive associations between income inequality and health. More research is needed to understand how income inequality drives health inequities in different ways on distinct geographic levels.

### Strengths and weaknesses

Two strengths of this study are that it is the first, to our knowledge, to directly compare the relationships between income inequality and population health by geographic level in the US, and the analysis specifically focused on income inequality’s impact on older adults. The factors that may influence population health in other age groups may be distinct from those that influence population health among older adults. Study findings provide evidence that the geographic level of measurement of inequality matters for population health. Other study strengths include the use of a large, nationally representative sample of older adults and looked at multiple diverse measures of population health and other health behaviors.

The results of this study should be interpreted with several important limitations. First, this analysis did not include a geospatial statistical component. Spatial autocorrelation may impact the standard error of model parameter estimates, since states and counties in close proximity to each other are more likely to share sociocultural and environmental characteristics than areas further apart. In the US, patterns of social, behavioral and economic influences on health and disease cluster by multi-state region, adding a third potential level of aggregation for future analyses. Second, this analysis is ecological and cross-sectional therefore causality cannot be assessed. Third, although more recent data are available, the analysis uses self-reported data collected in 2012, the last year in which county-level information is available for the entire contiguous US. The BRFSS sample itself is limited to community-dwelling individuals who are accessible by telephone (mobile or landline). Although the majority of US residents had telephone access [65] as of 2012, our analyses do not capture persons over 65 who no longer live independently, or are too disadvantaged to have telephones. These groups may have been especially impacted by income inequality across their lifetimes, in which case our findings may be conservative as to inequality’s effect on health in older age. Next, the analysis attempted to control for compositional differences between geographic areas and individuals: however, it was not possible to control for all possible confounders. Therefore, study results may be biased due to residual confounding by unmeasured variables or variables not included in the models. Relatedly, for the outcome of diabetes status, it is unknown whether the respondents who reported diabetes had type 1 or type 2 diabetes. This issue has particular importance in social determinants research because type 2 diabetes is often heavily influenced by behavioral factors. Type 2 diabetes is characterized by insulin resistance, whereas Type 1 diabetes is often genetic and occurs when the pancreas produces little or no insulin. Social determinants, such as the ones explored in this study, may promote or reduce the risk of developing type 2 diabetes in contrast to type 1 diabetes [66]. Another important limitation to consider is that, although five representative, diverse health outcomes and behaviors were examined, successful aging requires substantially more than these five health outcomes and behaviors. Future analyses can examine other, related factors, such as health care access, comorbidities, and other critical health behaviors. Future studies in the US can also examine the potential for local associations to occur, whereby certain health outcomes may be associated with income inequality on the local level, but the associations may not be evident when examining all counties or states at one time. Analogous multi-level methods could be applied in studies conducted in other countries, as well.

Another important caveat is the inherent heterogeneity of counties and states. It should be noted that counties and county-equivalent units vary enormously in both geographic and population size across the states and regions of the US, as well as by political function, and therefore the impact of county-level characteristics on population health likely also varies. With respect to variability in size, consider the case of Virginia: all independent cities, regardless of population size or land area, are considered to be county equivalents. As of the 2010 US Census, Norton, Virginia, a city with a population under 4000 and an area of approximately seven square miles would be considered to the a “county-equivalent unit”, equal to Fairfax County, with a population of over 1.1 million people and a land area of 396 square miles. The differences are more notable when comparing county sizes across the US. San Bernardino County, California is the largest in the US geographically with an area of 52,070 km^2^, which itself is over 16 times larger than area of the smallest entire state (Rhode Island, 3144 km^2^). For comparison, the smallest county in the US is Falls Church, Virginia—another county-equivalent unit—with a land area of just 5.4 km^2^. Washington, DC is another geographic anomaly. It would be considered a county-equivalent unit as well as a state-equivalent unit. New York City’s five boroughs are each considered county-equivalent units, yet some have populations themselves larger than that of US states [48]. Each of these examples is considered to be equivalent units of observation in an analysis of all US counties, despite their vast differences in geographic and population size.

There is substantial variability in terms of county function across the US, which may be partially attributable to variability in geographic and population size. Most US counties are the source of municipal governance, and provide services such as police and safety, vital records, taxation, and municipal services [67]. However, many New England states (e.g. Connecticut, Rhode Island, etc.) have no functioning county governments outside judicial districting. In those states, the municipalities (cities and towns) provide the services and perform the functions that counties do in most other US states [68]. Future studies could consider grouping adjacent counties of similar size and/or function for analytical purposes to reduce spatial heterogeneity among counties. Similar issues would arise in other countries, such as Australia, where there is substantial heterogeneity among LGAs, for example [29].

Lastly, one of the most important limitations of the analysis is the use of the Gini index as a measure of income inequality. Although the Gini index is the most widely used measure of income inequality in most biomedical and public health studies on this topic, a number of alternative measures on income inequality exist. Among its limitations, the Gini index is sensitive to inequalities in the center of the income continuum, and less sensitive to inequalities at the extreme ends of the continuum [69, 70], although there is some debate in the literature about this issue that deserves further study [42].

## Conclusion

The findings from this study suggest that the Swiss Paradox [27]—higher income inequality is associated with better health outcomes for some measures of population health—may exist for older adults in the US. Paradoxically, higher income inequality was associated with lower county-level rates of obesity, but not associated with state-level obesity rates. Higher income inequality was associated with higher rates of diabetes on the state level, which would be expected, but the same association was not evident not on the county level. It is clear that further research is required to understand the mechanisms behind the seemingly complex patterns of associations between income inequality and health in older adults. Although there is a preponderance of evidence that suggests that increasing income inequality is bad for health, it is essential to understand why those associations differ when examined on the small scale to maximize the effectiveness of any programs, interventions, or policies designed to mitigate the harmful impacts of high income inequality on health. These methodological issues can be extended to research in other countries, although each country has unique systems of geospatial aggregation. This exploratory study highlights the need to identify issues of this aggregation pertaining to measurement of social determinants to conduct meaningful and impactful research through which population health can be improved. Such research is particularly important for vulnerable older adults, who experience a greater impact of these inequalities on health and mortality.

## Data Availability

All study data are publicly available from the Centers for Disease Control and Prevention (CDC) and US Census Bureau.
